# Pink Cricket Club: Dramatic color change in a Neotropical leaf‐masquerading katydid (*Arota festae*, Griffini, 1896)

**DOI:** 10.1002/ecy.70333

**Published:** 2026-03-07

**Authors:** J. Benito Wainwright, Zeke W. Rowe, Matthew P. Greenwell, Patrick G. Cannon, Nathan W. Bailey, Graeme D. Ruxton

**Affiliations:** ^1^ Centre for Biological Diversity, School of Biology, University of St Andrews Fife UK; ^2^ Amsterdam Institute for Life and Environment, Vrije Universiteit Amsterdam Amsterdam The Netherlands; ^3^ School of Biological Sciences, University of Reading Reading UK; ^4^ Smithsonian Tropical Research Institute Panama City Republic of Panama

**Keywords:** animal coloration, color change, delayed greening, katydid, masquerade camouflage, Tettigoniidae

Camouflage is a widespread and well‐studied antipredator concealment strategy that allows animals to “hide in plain sight” (Cuthill, 2019). While the coloration of camouflaged animals is often fixed, some species can change color, permitting them to adjust their individual appearance to diverse backgrounds within their habitat (Stevens, 2016). The best‐known examples of this phenomenon are chameleons and cephalopod molluscs (squid, octopuses, and cuttlefish); but color changes can also occur at a much slower pace, usually via hormonal mechanisms, with shifts unfolding over hours, weeks, or even months (Duarte et al., 2017, 2025; Stuart‐Fox & Moussalli, 2009; Umbers et al., 2014). Gradual color change may be important for masquerading organisms that hinder identification by resembling specific objects—such as leaves, twigs, and stones—whose appearance can change slowly over time (Skelhorn, Rowland, & Ruxton, 2010), but examples of this are extremely rare. Here, we formally describe an illuminating color variation, within a single life stage, in the Neotropical katydid species (Orthoptera: Tettigoniidae) *Arota festae* (Griffini, 1896; subfamily: Phaneropterinae) from observations of a single individual, which upon discovery, was an intense hot pink color (Figure 1).

**FIGURE 1:**
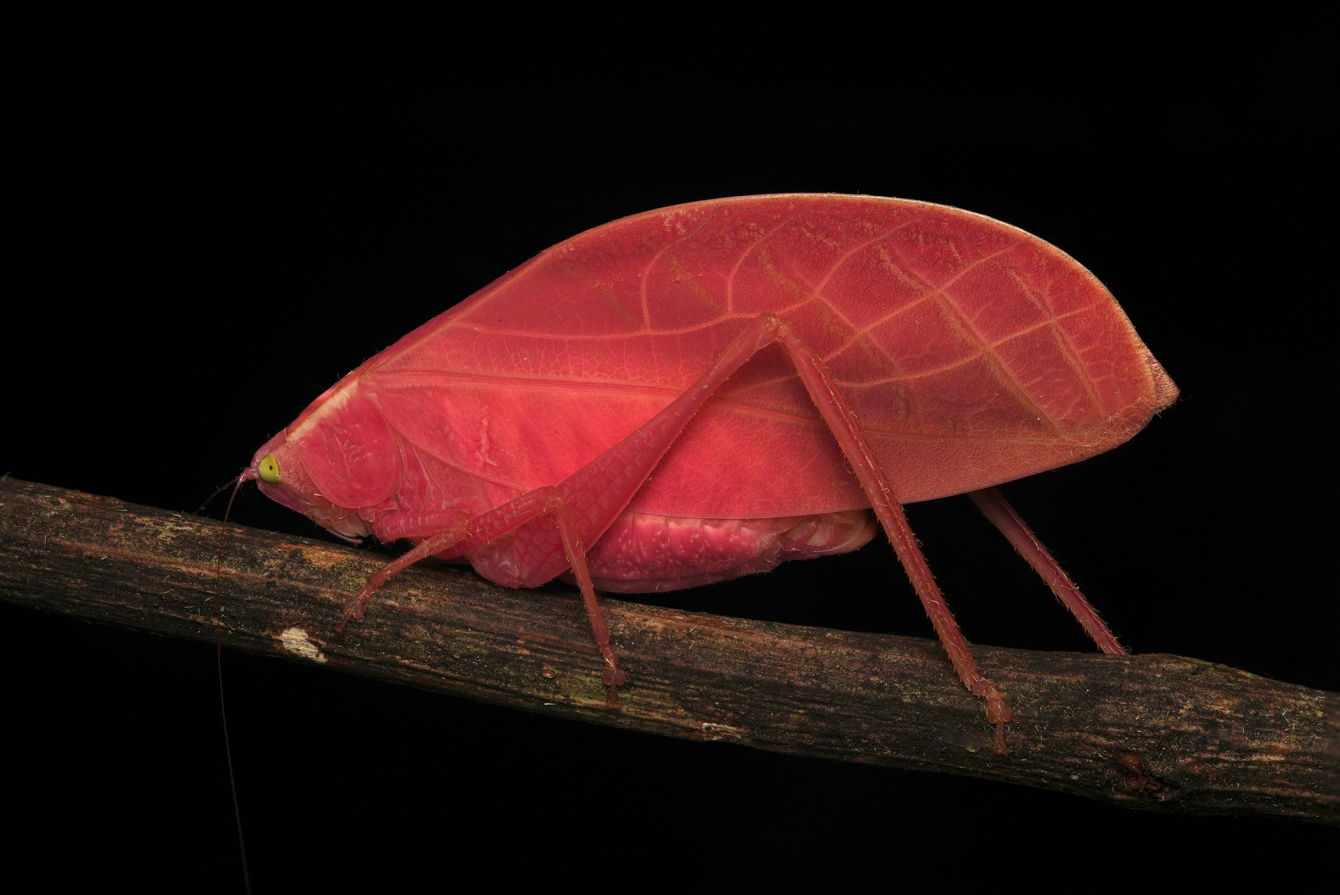
Intense hot pink morph of an adult female *Arota festae*. Photographed at 23:32 on 27 March 2025 on Barro Colorado Island, Panama, using a Sony A7CR camera with a LAOWA 90 mm f/2.8 lens and a Godox Speedlite TT350 flash. The final image was produced by focus stacking 4 photographs in Adobe Photoshop, with brightness increased for clarity while leaving saturation and hue unaltered. Photo credit: Zeke W. Rowe.

*A. festae* is a medium‐sized katydid (body length = ~27 mm; body mass = ~1 g; ter Hofstede et al., 2020), native to Panama, Colombia, and Suriname (Cigliano et al., 2020). It is typically a non‐sexually dimorphic light green color with broad, rounded forewings (tegmina) which generally resemble early growth vegetation. On 27 March 2025 at 23:12, an adult (i.e., final instar) female *A. festae* displaying an intense hot pink coloration was found on Barro Colorado Island (BCI), Panama (9.1647° N, 79.8367° W), underneath a research station light and within ~5 m of primary tropical rainforest (Figure 1, Figure 2A), where this species is relatively abundant (21 green individuals recorded during the same 4‐month field season). This individual was reared for 30 days at natural ambient temperature and humidity on BCI and fed ad libitum with mixed green vegetation, apple, and water in a 60 cm × 60 cm × 60 cm mixed species popup cage, which also included green *A. festae* morphs (the presence of a spermatophylax indicated that the pink morph successfully mated with one of these green individuals on Day 10; see Appendix S1: Figure S1). Green vegetation was haphazardly collected from the immediate surroundings and replaced every 2 days. After 4 days in captivity (31 March 2025), we noticed that the intensity of its pink hue had faded to a lighter pastel pink (Figure 2A). We subsequently monitored the coloration of this individual more closely by taking photographs every 24 h with the 12MP camera of an iPhone 13 Mini (Apple Inc., Cupertino, CA, USA). After a further 7 days, on 7 April 2025, this individual had turned completely green and was indistinguishable from individuals of the more common green morph (Figure 2A; Appendix S1: Figure S1). It remained this way until its natural death on 26 April 2025. In addition to this pink morph, and the more typical uniform green morph, we also discovered a previously unreported morph displaying patterning that resembles leaf necrosis. This morphotype is described and discussed in Appendix S1: Supplementary Text and illustrated in Figure S2.

**FIGURE 2:**
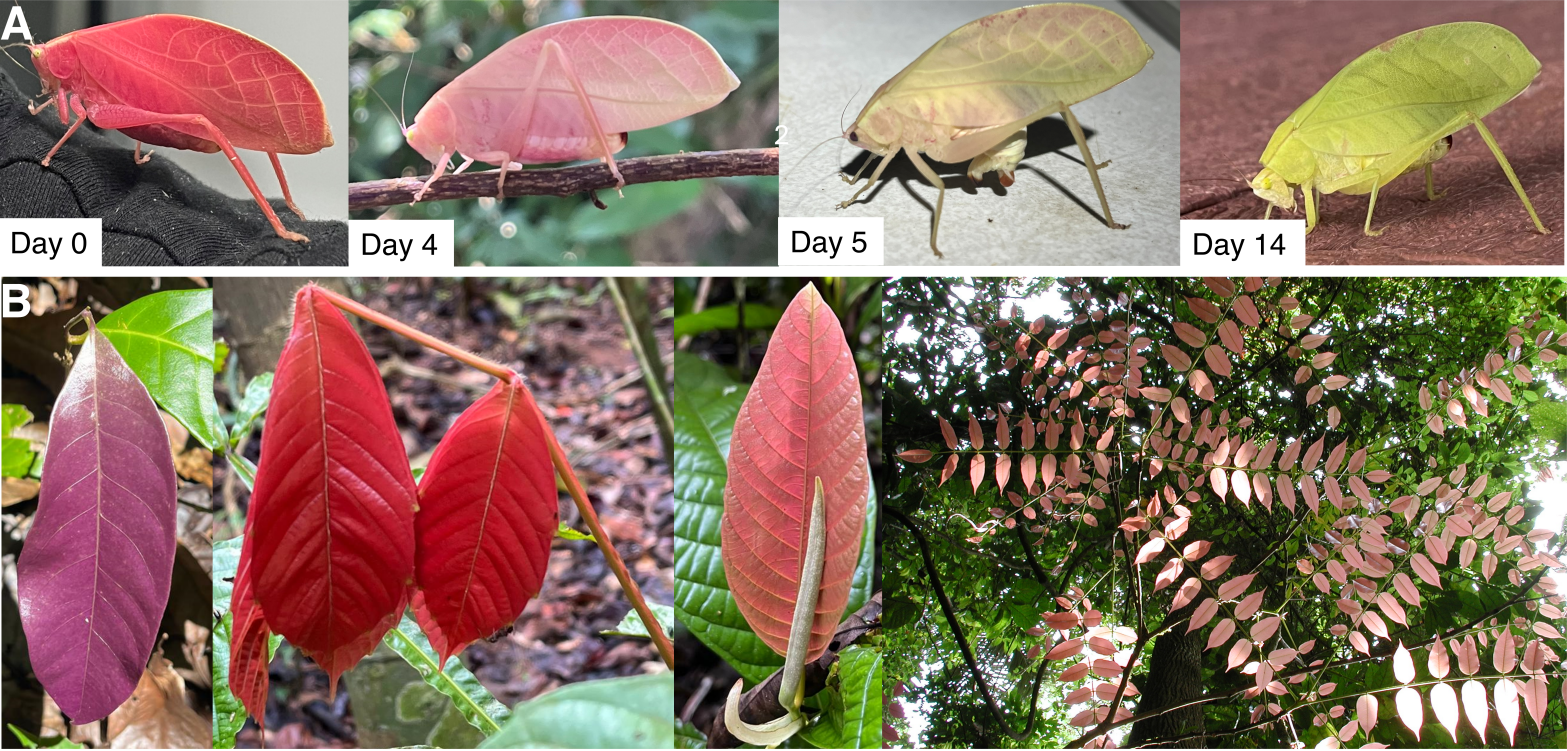
Pink‐to‐green color change in *Arota festae* (Griffini, 1896) and resemblance to pink leaves in delayed plant greening. (A) Photographs of the same *A. festae* individual at days 0, 4, 5, and 14 following initial discovery at 23:12 on 27 March 2025 on BCI, Panama. All photographs in (A) were taken by Benito Wainwright. (B) Photographs of local plant species displaying delayed greening (from left to right: *Paullinia bracteosa*, *Coccoloba manzinellensis*, *Inga ruziana*, and *Andira inermis*). From the left, the first, second, and third photographs in (B) were taken by J. Benito Wainwright, and the fourth photograph (on the far right in B) was taken by Phyllis Coley.

Homogenous pink coloration is extremely rare in insects, but in a limited number of studied cases from other katydid species, it has been thought to be the result of erythrism—a genetic mutation causing irreversible excessive red pigment production (Hancock, 1916; Ingrisch et al., 2016; Linn & Gillett‐Kaufman, 2015). While many tropical katydid species are known to resemble the color, shape, texture, venation, necrosis, and orientation of plant leaves (Castner & Nickle, 1995a; Wainwright et al., 2025), our observations provide an interesting example of color change in one such leaf‐masquerading species. Moreover, unlike many arthropods, which rely on ontogenetic changes to switch between camouflage strategies (e.g., Wade et al., 2008; Yu et al., 2021), this shift occurred within a single instar. Additional pink morphs exist for *A. festae*, and pink morphs are found in other katydid species such as *Paracyrtophyllus robustus*, *Amblycorypha oblongifolia*, and *Caedicia simplex*, which collectively span multiple families in Ensifera. The widespread occurrence of pink katydid morphs, despite their rarity within species, has been documented in the scientific literature since at least 1878 (Scudder, 1878; Wheeler, 1907), and strongly hints at an underlying ecological function. Our observation enables us to make testable predictions about the mechanistic basis and adaptive significance of this unusual trait based on current understanding of animal color change and katydid natural history.

Historically, pink coloration in katydids has been regarded as rare and disadvantageous (Hancock, 1916; Linn & Gillett‐Kaufman, 2015) due to the greater conspicuousness of these individuals to predators. However, the existence of a color change mechanism in adult *A. festae* hints that pink coloration might be functional in at least this species, but only under specific circumstances. The slow transition in morphological color observed suggests that the change is caused by the gradual degradation, chemical modification, or acquisition of pigments rather than from neuronal mechanisms (Duarte et al., 2025; Umbers et al., 2014), but the environmental cues controlling this process remain unidentified. It is unknown if the color change is reversible, or if the timing of the switch is genetically predetermined, but given the conditions in which our focal individual was raised, it is possible that diet (i.e., consumption of green vegetation in its rearing cage) and background color (i.e., resting on green vegetation in its rearing cage) contribute towards the transition. This would align with previously identified mechanisms underlying green–brown transitions in other cryptically colored cricket species (e.g., Lymbery, 1992; Umbers et al., 2014).

But what is the function of pink coloration, and is it an evolved adaptation? On BCI, 36% of plant species exhibit delayed greening, where young leaves display vivid colors ranging from white, red, to bright pink (also described as “red flushing”) due to reduced chlorophyll content (Coley & Barone, 1996; Gong et al., 2020; Kursar & Coley, 1992; Numata et al., 2004). Delayed greening is generally considered to function as an anti‐herbivory defense by decreasing the nutritional value and palatability of leaves, and the resulting leaf colors closely match those observed in *A. festae* over the course of its color change (Figure 2). Delayed greening is also widespread throughout the world's tropics, especially among shade‐tolerant tree species (Gong et al., 2020; Kursar & Coley, 1992). The lack of seasonality in these habitats means that red flushing leaves are present year‐round, albeit at much lower abundance than green leaves, offering reliable objects for local masquerading animals to resemble. DNA barcoding from digestive tract contents has previously shown that the generalist herbivorous diet of *A. festae* on BCI includes tree species (e.g., *Inga* spp.) that undergo delayed greening (Palmer et al., 2022). We thus propose that pink coloration in *A. festae* evolved to mimic the pink coloration seen in some tropical rainforest plants that show delayed greening, as this could provide antipredatory benefits both through background matching and masquerade (Skelhorn & Ruxton, 2011). The adaptive significance of the gradual nature of the katydid's color transition is an intriguing open‐ended question. It may simply be an emergent property of the underlying morphological color change mechanism rather than an evolved adaptive response. In visually cluttered tropical forests, where delayed greening leaves occur as a mosaic of developmental stages at any given time, a gradual color transition—particularly in a mobile insect—may be selectively neutral. However, where suitable leaf models are consistently available, it raises the question of why a color change mechanism of any sort would have evolved in this species.

Because they are less palatable to herbivorous insects (Lev‐Yadun, 2016), we do not expect *A. festae* to feed on delayed greening leaves, but delayed greening shoots do typically emerge from plant nodes adjacent to more nutrient‐rich, highly palatable green leaves. Despite being primarily nocturnal, *A. festae*, could therefore move short distances to feed or rest on green leaves during the day, while remaining camouflaged from visually guided predators (ter Hofstede et al., 2020). Although masquerading animals do not necessarily need to be resting against their background to avoid being recognized as profitable prey, the context in which this masquerade is viewed is known to be important for prey survival (Skelhorn, Rowland, & Ruxton, 2010; Skelhorn, Rowland, Speed, & Ruxton, 2010; Skelhorn & Ruxton, 2011). This context dependency could make a switch in individual appearance beneficial. For example, a green morph would be more effectively disguised than a pink morph when resting on an entirely green shoot, with all its delayed greening leaves fully matured. Predetermined gradual color change in delayed greening leaves could thus provide a scenario where gradual color change is beneficial, allowing *A. festae* to adjust its appearance to match the most abundant models in its immediate surroundings. Additionally, resting on or near pink leaves may reduce exposure to insectivorous predators that have learned to avoid this type of vegetation when foraging (e.g., as observed in McLellan et al., 2021). The relative rarity of within‐instar color changes across Orthoptera (Umbers et al., 2014) may indicate that such mechanisms are either more difficult to evolve than between‐instar color shifts or are only advantageous under specific ecological conditions, for example, when masquerading as specific leaf models that exhibit continuous variation in hue. Katydid nymphal instars lack fully developed wings and thus the pronounced leaf‐masquerading features of the adults (Castner & Nickle, 1995a, 1995b; Cigliano et al., 2020). Therefore, if *A. festae* gains protection by resembling delayed greening leaves, shifts in coloration between instars may not provide any advantage.

To determine if pink coloration in *A. festae* offers adaptive concealment in environments with delayed greening plants, population, ecological and life history data, alongside field‐based predation experiments, are required. If it does, this case study would provide a striking example of a polyphenism in a masquerading species, whereby individuals change color within their lifetime to align with the life stage of their “models.” The only studied cases we are aware of that examine color polyphenism in masquerade come from lepidopteran caterpillar species inhabiting temperate regions (e.g., Greene, 1989; Noor et al., 2008; Skelhorn & Ruxton, 2010). However, unlike *A. festae*, there is no evidence that these species can transition between morphs.

Resemblance to delayed greening leaves is not the only plausible explanation for the existence of pink coloration in *A. festae*; the mere novelty of the appearance of these morphs could trigger neophobic responses in predators, reducing their likelihood of attack. The presence of different color morphs within the same population might disrupt the search images predators use to find prey more efficiently (e.g., Pietrewicz & Kamil, 1979; Troscianko et al., 2021), but this strategy is only effective if pink morphs occur at low frequencies in a population, which is consistent with our observations of color variation in this species (Allen, 1988). An alternative possibility is that pink coloration is simply a non‐adaptive developmental by‐product, maintained by other forms of balancing selection. For instance, recessive pink genotypes might be selected against when expressed in homozygotes, but advantageous when expressed in heterozygotes, allowing them to persist in a population. However, limited evidence suggests that pink alleles are dominantly expressed in previously reported erythristic katydid species (Byrne, 1967; Hancock, 1916).

The color and pattern variation observed in *A. festae* (Appendix S1: Figure S2) has the potential to advance knowledge of adaptive color change in the natural world, and its role in optimizing camouflage through masquerade specifically. High species diversity, especially in the tropics, makes katydids well suited to explore these mechanistic and functional questions in future work, especially given pronounced within‐species differences observed in other leaf‐masquerading katydid lineages (e.g., those within the subfamily Pterochrozinae; Castner & Nickle, 1995a, Castner & Nickle, 1995b). Testing how these animals depend on specific habitat features will provide insights into the intricate web of interactions within tropical forest communities and illuminate how the struggle for survival has yielded such forms most flamboyant.

## CONFLICT OF INTEREST STATEMENT

The authors declare no conflicts of interest.

## Supporting information


Appendix S1.
