# Supplementary material for: Pink Cricket Club: Dramatic color change in a Neotropical leaf‐masquerading katydid (Arota festae, Griffini, 1896)
Source: Ecology. 2026 Mar 7;107(3):e70333. doi: 10.1002/ecy.70333 (PMC12966949; doi:10.1002/ecy.70333)
Supplement: Supplementary file 1 — Appendix S1. [file ECY-107-e70333-s001.pdf]

Appendix S1

Pink Cricket Club: Dramatic color change in a Neotropical leaf-masquerading katydid (Arota festae, Griffini, 1896). J. Benito Wainwright, Zeke W. Rowe, Matthew P. Greenwell, Patrick G. Cannon, Nathan W. Bailey, Graeme D. Ruxton. *Ecology.*

Supplementary text

In addition to the rare pink morphotype and the common green phenotype, we found three (out of 21) *A. festae* individuals of a previously unreported distinct green morph, characterised by transparent patches on the tegmen, bordered by brown pigmentation (Figure S2). Two males and one female were found. The number, size, and shape of these patches varied between the three individuals; however, unlike the pink morph, no changes in appearance were observed when reared under the same conditions. We therefore predict that this is likely a genetic or developmentally induced polymorphic trait, that improves leaf masquerade by resembling necrotic patches and holes commonly found on damaged or decaying leaves, as previously seen in some other insects, including katydids (Costello *et al.* 2020; Mugleston *et al.* 2016). Unlike the gradual pink-green transition of delayed greening leaves, leaf damage does not change predictably over time. As a result, leaf damage masquerade is unlikely to benefit from color change. *A. festae* may therefore represent a rare example of a species exhibiting both environmentally induced polyphenism and genetic polymorphism to enhance its masquerade camouflage, though substantial genetic, evo-devo, and physiological evidence is required to support this suggestion.

Supplementary figures

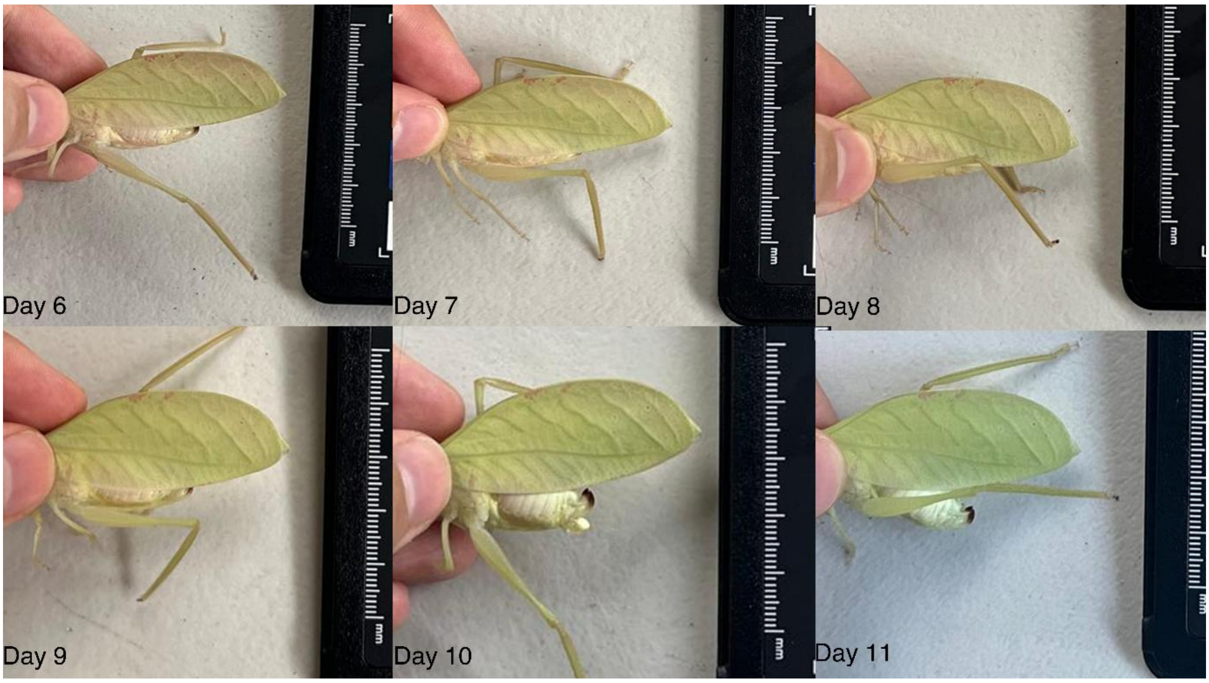

Figure S1. Chronological photographs of the same *Arota festae* (Griffini, 1896) individual, taken with a suitable scale under natural lighting on day 6, 7, 8, 9, 10 and 11 following initial discovery (27 March 2025) on BCI, Panama. Photos by J. Benito Wainwright.

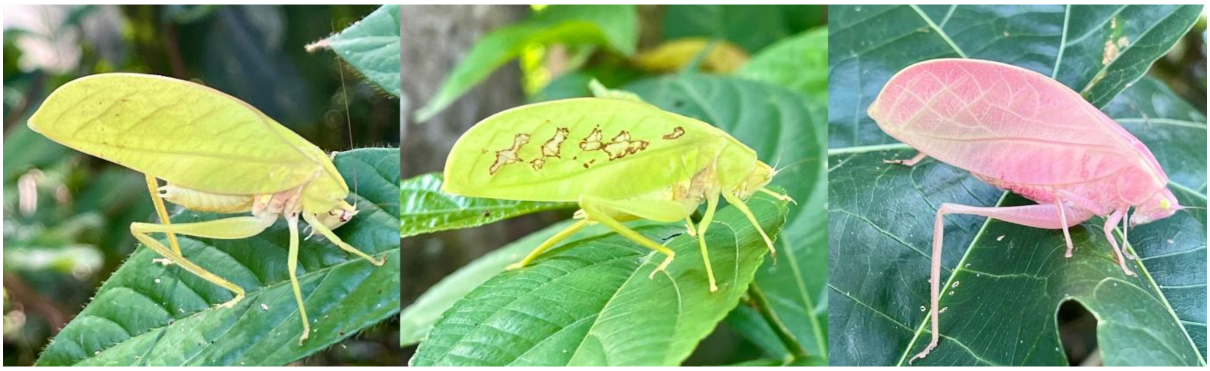

Figure S2. Positioned photographs of the three local *Arota festae* (Griffini, 1896) color morphs all taken on 31 March 2025 on BCI, Panama. From left to right: typical green, green necrotic, pink. Photos by J. Benito Wainwright.

Supplementary references

Costello, L. M., Scott-Samuel, N. E., Kjernsmo, K. & Cuthill, I. C. (2020). False holes as camouflage. *Proc R Soc B*, **287**, 20200126.

Mugleston, J., Naegle, M., Song, H., Bybee, S. M., Ingley, S., Suvorov, A. & Whiting, M. F. (2016). Reinventing the leaf: multiple origins of leaf-like wings in katydids (Orthoptera: Tettigoniidae), *Invertebr Syst*, **30**, 335-352.
